# Exploring Singapore’s consumption of local fish, vegetables and fruits, meat and problematic alcohol use as risk factors of depression and subsyndromal depression in older adults

**DOI:** 10.1186/s12877-019-1178-z

**Published:** 2019-06-10

**Authors:** Chong Min Janrius Goh, Edimansyah Abdin, Anitha Jeyagurunathan, Saleha Shafie, Rajeswari Sambasivam, Yun Jue Zhang, Janhavi Ajit Vaingankar, Siow Ann Chong, Mythily Subramaniam

**Affiliations:** Research Division, Institute of Mental Health, Buangkok Green Medical Park, 10 Buangkok View, Singapore, 539747 Singapore

**Keywords:** Depression, Risk factors, Singapore, Diet, Older adult, Fish, Vegetables, Fruits, Alcohol

## Abstract

**Background:**

Depression is a chronic mental disorder that severely impacts the older adult population globally. Nutritional psychiatry is an approach that has gained traction over the years. Exploring locally relevant consumption of common types of fish, vegetables and fruits (V&F), meat and problematic alcohol use (PAU) as risk factors associated with depression and subsyndromal depression (SSD) could reveal modifiable factors that could be targeted in the local older adult population in Singapore.

**Methods:**

Data collected from the Well-being of the Singapore Elderly (WiSE) study, a cross-sectional population-based epidemiological study of Singapore’s older adult population was analysed for the purposes of this study. Two thousand five hundred sixty-five participants were recruited and comprised of Singapore citizens and permanent residents aged ≥60 years. Data on fish, meat, and V&F consumption were collected using the sociodemographic and risk factor questionnaire. The CAGE (Cut, Annoyed, Guilt, and Eye-opener) questionnaire was used to determine PAU. The Geriatric Mental State-Automated Geriatric Examination for Computer Assisted Taxonomy (GMS-AGECAT) was used to obtain participants’ diagnosis of depression or SSD. A multinomial logistic regression was used to explore the relationship between depression and dietary factors.

**Results:**

Consumption of V&F in the last 3 days was less likely to be associated with depression and SSD. Frequent consumption of specific species of fish was associated with depression and SSD. PAU and the frequent consumption of *Himantura gerrardi* (stingray) were more likely to be associated with SSD. Finally, meat consumption was more likely to be associated with depression and SSD.

**Conclusions:**

The preliminary findings of the study support a need for healthy eating for the older adult population in Singapore. Further directions include a more thorough health and nutrition survey to capture accurate diets among the older adults in Singapore.

## Background

Depression is a chronic mental disorder that negatively impacts an individual’s affect, cognition and behaviour [1]. According to the World Health Organisation (WHO) report based on 2015 data, the global prevalence of depression was estimated at 4.4% of the world’s population. The report further found that the global prevalence of depression peaked among older adults aged 55 to 74 years [1]. Valiengo et al. reported that unipolar depression occurred in 10% to as high as 38% of the older adult population [2]. In comparison to their younger counterpart, the impact of depression on the older adult population is more severe; and results in greater associations with negative outcomes, such as medical (i.e. psychiatric and physical) comorbidities, the increased risk of suicide and above all else, increased mortality rates [2]. The world’s ageing population was declared by the United Nations (UN) as a global issue with a projected exponential increase to approximately 2.1 billion by 2050 [3]. As the life expectancy of the world continues to rapidly increase, research is necessary to ensure a physically, and mentally healthy ageing population.

In line with the increasing trend of an aging population worldwide [3] a similar trend is observed in Singapore’s population where in 2017 the median age was 40.0 years, and those ≥60 years comprised of 19.5% of Singapore’s population. It is further reported that by 2050, 40.1% of Singapore’s population would comprise older adults aged ≥60 years. The Singapore Mental Health Study (SMHS) found major depressive disorder to be the most prevalent mental disorder in Singapore with a lifetime prevalence of 5.8% [4]. In addition, according to the WHO report for depressive disorders, Singapore contributed up to 9% of total years lived with disability and was ranked third in terms of prevalence (4.6% of the population) within the Western Pacific region. However, the Well-being of the Singapore Elderly (WiSE) study found that the prevalence of depression cases among older adults in Singapore had decreased by 1.8% in the last decade [5]. Therefore, exploring locally relevant factors associated with depression could identify putative modifiable factors that could be potentially targeted in the older adult population.

Risk factors of depression are complex and multifactorial. Research has explored biological [6, 7], and psychosocial [8] approaches, which in turn contributed to the plethora of literature on understanding the influences that could exacerbate the onset of depression. An approach that has gained popularity within the psychiatric community is the study of diets and its association with mental illnesses [9, 10]. For instance a study in Australia found that diets with low intake of healthy food or high intake of unhealthy food, based on the food frequency questionnaire (FFQ), were associated with an increased risk of depression among older adults over time [9]. Additionally consumption of meat, which is a crucial component of a typical diet, had been associated with depression in a meta-analysis [11]. Mediterranean [12, 13], Japanese [7, 14], and Norwegian [15] diets and their association with depression had also been studied. In the UK, studies have established that high levels of alcohol use could lead to an increased risk of depression [16, 17]. In 2012, Caputo et al. reported that regular consumption of alcohol was seen among approximately 50% of the older adult population aged over 65, and 25% for over 85 years of age [18]. In Singapore, 10.1% of the older adult sample from the WiSE study was found to have a lifetime drinking problem [19]. As alcohol is readily available in Singapore, problematic alcohol use (PAU) in the older adult populations requires careful consideration.

On the other hand, there are components of a diet that have been suggested to have protective properties [20–23]. The consumption of fish had been identified as a protective factor against depression [20, 21, 24, 25]. Studies have suggested that docosahexaenoic acid (DHA) and eicosapentaenoic acid (EPA), commonly known as omega-3 oil or fish oil possess certain neurobiological properties that protect against depression [6, 20]. This was further supported by a meta-analysis that indicated high intake of fish resulted in reduced risk of depression [21]. Although, a systematic review among Japanese older adult sample found that only moderate intake of fish was associated with lower risk of depression [20]. There are studies that have explored fatty acids profiles of several different fish [26–31], but limited research has been done on their association with depression. In addition, the consumption of vegetables and fruits (V&F) has an established association with the risk of depression [22, 23]. It is suggested that the beneficial bioactive compounds found in V&F provides the protective component against depressive symptoms [23, 32–34]. Wolniczak et al. and Bishwajit et al. found an inverse association, i.e. individual’s whose diet comprised of less V&F were more likely to exhibit depressive symptoms [22, 23].

Singapore has a stable supply of imported goods from across the globe, which provides the residents access to a wide range of food [35]. As a major consumer of fish and seafood products; the World Wide Fund for Nature (WWF) reported that the average citizen in Singapore consumed approximately 22 kg of fish and seafood per capita in 2017, which attests to 10% more than the global average per capita (20 kg) [36]. Additionally, food such as vegetables (i.e. leafy, roots, etc.), fruits, and meat are also imported from countries such as the United States, Australia, and Netherlands [35]. In 2014, the WHO reported an increase of 1 l of pure alcohol per capita consumption in 2008–2010 compared to 2003–2005 in Singapore. Although the trending patterns indicated a risk score of only 2 (1 being the least risky, and 5 being the most); in 2010 alone, the average of pure alcohol per capita consumption almost doubled (i.e. 4 l) [37]. Therefore, this paper aims to explore the association between depression and SSD among older adults and: (a) the common types of frequently consumed fish; (b) the consumption of vegetables and fruits within the last 3 days; (c) problematic alcohol use; and (d) the consumption of meat in Singapore.

## Methods

### Study overview

Data collected from the Well-being of the Singapore Elderly (WiSE) study completed in December 2013 was analysed for the purposes of this study. The WiSE study was a cross-sectional population-based epidemiological household study that involved the collection of sociodemographic information, physical, cognitive and mental status of older adults. Approval for the study was granted by the institutional ethics committee: the National Healthcare Group, Domain Specific Review Board, and the Singhealth Centralised Institutional Review Board. Written informed consent was obtained from the participants preceding the survey and the examination. In the event of participants who were unable to provide informed consent, written informed consent was ascertained from their legally acceptable representative or next of kin. A prior article provides detailed methodology of the WiSE study [38]. The study adopted the widely validated 10/66 protocol for this specific study population [38].

### Study participants

The WiSE study comprised of Singapore citizens and permanent residents aged ≥60 years (mean 72.7, SD = 9.53, range 60 to 105) [39] living in Singapore at the time of the survey. An administrative national database of Singapore residents was utilised for random selection of participants. This also included residents who were residing in nursing homes, institutions and day care centres in Singapore during the time of the survey. Residents who were not living in Singapore and uncontactable at the time of the survey were excluded from the study. The study recruited a total of 2565 participants with disproportionate random sampling to ensure appropriate proportions of Singapore’s prominent ethnic groups: Chinese (*n* = 1012, 39.5%), Malay (*n* = 745, 29.0%), Indians (*n* = 772, 30.1%), and other ethnicities (*n* = 36, 1.4%).

### Measures & outcomes

#### Fish consumption

As part of the sociodemographic and risk factor questionnaire, participants were asked “*How often do you eat fish?”* where possible answers included: “never”, “some days”, “most days”, and “every day”. For the purpose of this study, “every day”, “most days” and “some days” were combined into a single category (i.e. yes). Participants who answered other than “never” were asked “What type of fish do you usually eat?” Possible answers included: “Seabass” (*Lates calcarifer*), “Stingray” (*Himantura gerrardi*), “Grouper” (*Epinephelus coioides*), “Pomfret” (*Pampus chinesis*), “Yellow Tail” (*Atule mate*), and “Others, specify”. After extracting from “Others, specify” category, the 2 most frequent fish that were mentioned included: “Snapper” (*Pristipomoides multidens*) and “Mackerel” (*Scomberomorus commerson*).

#### Meat consumption

Participants were asked “*How often do you eat meat?*” where possible answers included: “never”, “some days”, “most days”, and “every day”; the answers were then stratified into whether the participants did or did not consume meat overall.

#### Vegetables and fruits (V&F) consumption

Participants were similarly asked to provide information on “*How many servings of fruit and vegetables have you eaten over the last 3 days?*” where 1 serving was defined as 1 fruit or 1 portion of salad/ vegetables. This study extracted the data, and stratified it into whether the participants did not or did consume V&F over the last 3 days.

#### The CAGE questionnaire

The WiSE study used the CAGE (Cut, Annoyed, Guilty, Eye-opener) self-report questionnaire to assess the participants’ alcohol use status. It has 5 questions – Question 1 enquired “*Was there ever a period in your life when you drank at least 12 drinks in a* year” Possible answers include: “I never drank alcoholic drinks”, “No (less than 12 drinks per year)”, “Yes”. If participants answered “Yes” to consumption of more than 12 alcoholic drinks per year they proceeded to answer the subsequent questions. Question 2 asked “*Have you ever felt you should cut down on your drinking*”; Question 3 asked “*Have people annoyed you by criticizing your drinking?*”; Question 4 enquired “*Have you ever felt bad or guilty about your drinking*”; Question 5 asked “*Have you ever had a drink first thing in the morning to steady your nerves or to get rid of a hangover (eye opener)?*”. Possible answers for questions 2 to 5 were no or yes. The answers were then tallied for a possible score of 0 to 4; where the cut off score for problematic alcohol use was 2 [40].

#### Socioeconomic and demographic covariates

Age was grouped into “60–74”, “75–84”, and “> 85” in years. Sex was divided into “Male” and “Female”. Ethnicity included “Chinese”, “Malay”, “Indian”, and “Others”. The marital status of the participants was grouped into “Married/Cohabitating”, “Never Married”, “Widowed”, and “Divorced/Separated”. Education categories included “None”, “Some, but did not complete primary”, “Primary”, “Secondary”, and “Tertiary” with tertiary education defined as having at least completed polytechnic/junior college or equivalent (i.e. college) and up to university. The employment status of the participants was grouped into “Employed”, “Homemaker”, and “Retired”. The number of household assets (“Cars”, “Television”, “Fridge/Freezer”, “Telephone/Mobile Phone”, “Air Conditioning”, “Paid TV”, “Computer” and “Washing Machine”) was based on the sum of their assets, and for the purpose of the regression analysis and skewed data, it was stratified into 5 ordinal-level categories based on the sum of their assets (0–3 into 1, 4–5 into 2, 6 into 3, 7 into 4, and 8 into 5); where being in a higher category would suggest a higher socioeconomic status. Body mass index (BMI) was estimated using weight and height measured by the field interviewers. Smoking status included “Never Smoked”, “Smoked Before”, and “Current Smoker”. Physical activity was measured using the question “*Taking into account both work and leisure, would you say that you are*”. Possible answers included 1 to 4, which represented “very physically active” as 1, “fairly physically active” as 2, “not very physically active” as 3 and “not at all physically active” as 4. For the purpose of the paper, answers 1 to 2 were combined into a single category (i.e. yes), whereas answers 3 to 4 were combined into “no”.

#### Depression and Subsyndromal depression

The Geriatric Mental State (GMS) was utilized in the WiSE study as a semi-structured interview with the Automated Geriatric Examination for Computer Assisted Taxonomy (AGECAT) to obtain the participants’ diagnoses. The GMS-AGECAT generated 4 syndrome clusters: depression; anxiety neurosis; dementia; schizophrenia and related paranoia. Each syndrome cluster was then given a severity rating ranging from 0 (no symptoms) to 5 (very severe). Participants who scored 3 or higher in the Depression cluster were considered “cases” and classified as “Depression”. A score of 1 or 2 placed the participants under the classification of “Subsyndromal Depression” (SSD) [5]. As indicated by Subramaniam et al. [38], the GMS-AGECAT has been widely utilised as a diagnostic instrument for the older adult population, and has been validated for Singapore’s older adult population [41].

### Statistical analysis

Statistical analysis was performed using STATA version 13. All estimates were weighted to ensure that the findings were representative of the 2011 older adult population in Singapore. Descriptive analyses were used to calculate the frequency and percentage for categorical variables. Multinomial logistic regression analysis was used to explore the relationship between depression and the consumption of common types of fish, meat and V&F; after adjusting for sociodemographic variables and health status (i.e. smoking). Depression status had three categories (depression, SSD, and non-depression; the non-depression group was used as a reference category). The multinomial logistic regression analysis was conducted based on complete (listwise deletion) analysis. Statistical significance was evaluated at the *p* value < 0.05 level using 2-sided tests.

## Results

### Sample description

A total of 2565 older adult participants completed the WiSE study. Majority of the older adults were aged 60–74 (75.0%), female (55.9%), of Chinese ethnicity (83.3%), either married or cohabitating (64%) and retired (38.3%). Table 1 presents the sociodemographic profile of the WiSE sample. The most commonly consumed fish in Singapore were: *P. chinesis* (53.1%), *E. coioides* (41.9%), *S. commerson* (40.9%), *A. mate* (37.6%), *L. calcarifer* (33.4%), *P. multidens* (27.6%), and *H. gerrardi* (22.6%).

**Table 1:** Sociodemographic Profile of the WiSE Sample

Variable	Category	*n*	Unweighted %	Weighted %
Age (in years)	60–74	1494	58.3	75.0
	75–84	669	26.1	19.5
	85+	402	15.7	5.5
Sex	Male	1117	43.6	44.1
	Female	1448	56.5	55.9
Ethnicity	Chinese	1012	39.5	83.3
	Malay	745	29.0	9.3
	Indian	772	30.1	6.0
	Others	36	1.4	1.4
Marital Status	Married/Cohabiting	1484	57.9	64.0
	Never Married	136	5.3	8.0
	Widowed	836	32.6	22.5
	Divorced/Separated	107	4.2	5.5
Education	None	511	20.0	16.5
	Some, but did not complete primary	620	24.3	23.9
	Primary	640	25.1	24.8
	Secondary	517	20.3	22.4
	Tertiary	262	10.3	12.4
Employment Status	Employed	688	27.2	33.9
	Unemployed	32	1.3	1.5
	Homemaker	808	31.9	26.3
	Retired	1006	39.7	38.3
Smoking Status	Never Smoked	1886	73.7	74.5
	Smoked Before	439	17.1	15.9
	Current Smoker	236	9.2	9.5
GMS-AGECAT	Depression	177	6.9	3.73
	Subsyndromal Depression	425	16.6	13.4
	Non-depression	1963	76.5	76.5
Seabass (*L. calcarifer*)	No	1591	65.9	66.7
	Yes	824	34.1	33.4
Stingray (*H. gerrardi*)	No	1698	70.3	77.4
	Yes	717	29.7	22.6
Grouper (*E. coioides*)	No	1613	66.8	58.1
	Yes	802	33.2	41.9
Pomfret (*P. chinesis*)	No	1248	51.7	46.9
	Yes	1167	48.3	53.1
Yellow Tail (*A. mate*)	No	1408	58.3	62.4
	Yes	1007	41.7	37.6
Snapper (*P. multidens*)	No	1814	70.7	72.4
	Yes	751	29.3	27.6
Mackerel (*S. commerson*)	No	1452	56.6	59.1
	Yes	1113	43.4	40.9
Vegetables and Fruits	No	46	1.81	1.4
	Yes	2493	98.2	98.6
Meat	No	193	7.6	3.3
	Yes	2345	92.4	96.7
Problematic Alcohol Use	No	2464	96.1	95.8
	Yes	101	3.9	4.2
Physically Active	No	871	33.96	24.5
	Yes	1694	66.04	75.5
Variable		*n*	Unweighted (*mean*)	Weighted (*mean*)
Body Mass Index (BMI)		2289	25.10	24.49
Household Assets		2547	6.28	6.38

### Diet and problematic alcohol use

A multinomial logistic regression analyses (Table 2) was used and revealed statistical significance for frequent consumption of *L. calcarifer* (*p* < .05), *P. multidens* (*p* = .05), and *H. gerrardi* (p < .05), V&F consumption within the last 3 days (*p* < .001), meat (p < .05; p = .05) and PAU (p < .05). The consumption of V&F within the last 3 days was less likely to be associated with depression (OR: 0.11; 95% CI: 0.02, 0.45) and SSD (OR: 0.10; CI 95%: 0.03, 0.39). The frequent consumption of *L. calcarifer* (OR: 0.58; 95% CI: 0.36, 0.95) was less likely to be associated with SSD, and the frequent consumption of *P. multidens* (OR: 0.54; 95% CI: 0.29, 1.00) was less likely to be associated with depression. The frequent consumption of *H. gerrardi* (OR: 1.91; 95% CI: 1.20, 3.04) was more likely to be associated with SSD. Meat consumption was more likely to be associated with depression (OR: 3.21; 95% CI: 1.02, 10.14) and SSD (OR: 2.61; 95% CI: 1.20, 5.67). PAU (OR: 2.81; 95% CI: 1.25, 6.33) was more likely to be associated with SSD.

**Table 2:** Sociodemographic, Fish, Vegetables and Fruits, Meat, and Problematic Alcohol Use Correlates of Depression and Subsyndromal Depression

		Depression^a^				Subsyndromal^a^			
Variables	Category	OR	95% CI		*p*	OR	95% CI		*p*
Diet	Seabass (*L. calcarifer*)	0.67	0.39	1.16	0.15	0.58	0.36	0.95	0.03
	Stingray (*H. gerrardi*)	1.31	0.71	2.45	0.39	1.91	1.20	3.04	0.01
	Grouper (*E. coioides*)	0.72	0.38	1.35	0.31	0.83	0.53	1.30	0.41
	Pomfret (*P. chinesis*)	1.68	0.89	3.16	0.11	0.92	0.60	1.41	0.69
	Yellow Tail (*A. mate*)	1.24	0.68	2.26	0.49	1.34	0.92	1.95	0.13
	Snapper (*P. multidens*)	0.54	0.29	1.00	0.05	0.80	0.53	1.21	0.30
	Mackerel (*S. commerson*)	0.88	0.51	1.52	0.66	1.30	0.89	1.90	0.18
	Vegetables & Fruits	0.11	0.02	0.45	0.00	0.10	0.03	0.39	0.00
	Meat	3.21	1.02	10.14	0.05	2.61	1.20	5.67	0.02
Problematic Alcohol Use		2.04	0.62	6.67	0.24	2.81	1.25	6.33	0.01
Age (in years)	60–74	Ref.				Ref.			
	75–84	1.88	0.94	3.75	0.07	0.85	0.53	1.36	0.49
	85+	1.92	0.69	5.33	0.21	1.08	0.59	2.00	0.80
Sex	Male	Ref.				Ref.			
	Female	2.33	0.79	6.84	0.13	1.41	0.82	2.42	0.22
Ethnicity	Chinese	Ref.				Ref.			
	Malay	2.36	1.18	4.72	0.02	0.89	0.59	1.34	0.57
	Indians	4.99	2.71	9.18	0.00	1.71	1.18	2.47	0.01
	Others	2.07	0.27	15.86	0.48	1.32	0.49	3.56	0.58
Marital status	Married/Cohabitating	Ref.				Ref.			
	Never married	0.47	0.08	2.62	0.39	1.23	0.59	2.57	0.58
	Widowed	0.47	0.22	1.02	0.06	1.22	0.76	1.95	0.42
	Divorced/Separated	0.78	0.32	1.88	0.58	2.17	0.97	4.87	0.06
Education	Tertiary	Ref.				Ref.			
	None	0.85	0.22	3.22	0.81	1.83	0.81	4.15	0.15
	Some, but did not complete Primary	0.42	0.12	1.50	0.18	1.43	0.68	3.00	0.34
	Primary	0.69	0.21	2.22	0.53	0.92	0.43	1.96	0.84
	Secondary	0.49	0.16	1.55	0.23	1.22	0.58	2.54	0.60
Employment Status	Employed	Ref.				Ref.			
	Unemployed	1.35	0.22	8.46	0.75	1.74	0.30	10.07	0.54
	Homemaker	1.00	0.39	2.56	0.99	1.01	0.58	1.77	0.96
	Retired	1.08	0.49	2.35	0.86	0.93	0.58	1.51	0.77
Smoking Status	Never Smoked	Ref.				Ref.			
	Smoked Before	0.82	0.29	2.31	0.71	1.08	0.63	1.85	0.79
	Current Smoker	1.53	0.49	4.82	0.47	0.59	0.30	1.13	0.11
Household Assets		0.90	0.74	1.10	0.31	0.91	0.79	1.04	0.17
Physically Active	No	Ref.				Ref.			
	Yes	0.20	0.11	0.36	0.00	0.56	0.36	0.86	0.01
Body Mass Index (BMI) (*mean*)		1.02	0.96	1.08	0.51	1.01	0.98	1.05	0.51

^a^Non-depression group was used as the reference category

### Sociodemographic correlates

Malay (OR: 2.36; 95% CI: 1.18, 4.72; *p* < .05) and Indian (OR: 4.99, 95% CI: 2.71, 9.18; *p* < .001) ethnicity were more likely than Chinese to be associated with depression. Additionally, the Indian ethnicity was also more likely to be associated with SSD (OR: 1.71; 95% CI: 1.18, 2.47; p < .05). Engaging in physical activity compared to no physical activity was less likely to be associated with depression (OR: 0.20; 95% CI: 011, 0.36; *p* < .001) and SSD (OR: 0.56; 95% CI: 0.36, 0.86; *p* < .05).

## Discussion

### Fish consumption

The fish consumption in Singapore suggests that there are certain components that could influence an individual’s association with depression and subsyndromal depression (SSD). As shown by the results, the frequent consumption of *L. calcarifer* was less likely to be associated with SSD and the frequent consumption of *P. multidens* was less likely to be associated with depression. Whereas frequent consumption of *H. gerrardi,* was more likely to be associated with SSD. A recent meta-analysis reported the neuro-benefitting properties of polyunsaturated fatty acids (PUFA) as a protective factor against the development of depression [21]. PUFA are found naturally in fish, and their fatty acid profiles could play a role in the development of depression. It had been reported that PUFA could be involved in the structural development and functional features of the brain, as well as having anti-depressant properties [6, 20]. Studies have shown there are significant differences in levels of PUFA between different types of fish [27–31]. A possible explanation for the significant difference between *L. calcarifer*, *P. multidens* and *H. gerrardi* could be the level of PUFA intake by the older adult population. To the best of the author’s knowledge, there have not been detailed studies on the fatty acid profiles of *H. gerrardi*, however, it had been previously reported that stingrays have relatively lower PUFA content compared to *P. multidens* [31] and *L. calcarifer* [27] from studies that examined other species of stingrays i.e. *Mobula birostris*, *Dasyatis sabina*, and *Menticirrhus americanus* [42, 43]. Thus, older adults in Singapore who frequently consume *H. gerrardi* as their primary source of “fish” intake may not be receiving sufficient levels of PUFA in their diet. However, the study did not control for other food consumption that were rich in PUFA such as legumes, nuts, cereals and oils [44]. Upon further examination, similar fish that were studied: *E. coioides*, and *S. commerson* were found to have higher PUFA profiles than *L. calcarifer* but did not achieve any statistical significance [31]. Within the limitations and scope of the study, it was not possible to explore the extent of PUFA protective properties and its influence on depression and SSD. Perhaps the interactions and interplay of additional nutritional properties of fish are involved, and have yet to be considered. Therefore, detailed investigation into the different fatty acid profiles and nutrients of the common fish found in Singapore could provide locally relevant insights to help inform future studies.

### Vegetables and fruits consumption within the last 3 days

Those who consumed V&F in the past 3 days showed lower probability of depression and SSD. Prior studies support these findings, and had reported that consumption of V&F decreased the risk of depression [22, 23, 32–34]. The bioactive compounds found in V&F are suspected to play a protective role against depression and depressive symptoms. Studies have typically reported lower levels of these bioactive compounds (i.e. vitamin c, antioxidants and ß-cryptoxanthin) among the older adult population [33, 34]. Wolniczak et al. and Bishwajit et al. found an inverse association, where low consumption levels of V&F (< 5 servings per day) were associated with a higher risk of depressive symptoms and behaviour [22, 23]. The WHO recommended at least 400 g (≥5 servings) consumption of V&F per day [22]; which estimates the total to 146 kg per capita of V&F in a year. According to a 2018 report by the Agri-Food and Veterinary Authority of Singapore, 94 kg of leafy and other vegetables, and 70 kg of fruits were consumed per capita for the fiscal year of 2017 [35]; which indicates that the average resident of Singapore (including PRs and foreigners) consumed higher rates of V&F per capita than that was recommended by the WHO. Although the report included foreigners, nevertheless the high consumption rates of V&F could possibly be one contributing factor for the relatively low prevalence of depression among the older adults in Singapore.

### Problematic alcohol use

In this study, Singapore’s older adult population with PAU were more likely to present with SSD. The association of increased alcohol intake and depression is strongly supported by Awaworyri et al. [16], and Boden et al. [17]. The increased likelihood of SSD could be explained by the psychoneuroimmunological approach [45, 46]. Immune cell modifications have been observed in the central nervous system due to the structural and physiological changes as the brain ages [47]. Damage and neuronal death could lead to vulnerabilities to inflammations and cognitive decline for the older adult [48]. Additionally, high levels of alcohol use could lead to immunomodulation effects such as inflammation [46]. Research has indicated that neuro-inflammation could invoke depressive episodes and the development of depression [45]. Paired with the inflammatory vulnerabilities that come with ageing, it could explain the increased likelihood for SSD with PAU. Alternatively, being depressed could potentially lead to problematic alcohol use among older adults in Singapore [17].

### Meat consumption

There was a significant association between depression and SSD and the consumption of meat. Meat consumption contributes to a large portion of dietary intake of an average individual, and has previously been associated with depression [11, 49]. One possible explanation for the greater association between depression and SSD could be the higher levels of trans-unsaturated fats (TFA) found in meat such as beef, chicken and mutton [50, 51]. According to Sanchez-Villegas et al. [50], TFA has a detrimental effect on depression risk, and the authors drew similarities of the mechanics to TFA and its influence on cardiovascular disease risk. For instance, TFA has been associated with some of the detrimental modifications in endothelial cell signalling pathways and pro-inflammatory cytokines, which has previously been reported to be lower in individuals with depression. Furthermore, TFA and its association with higher risk of depression has been reported in other studies [11, 51]. A common practice by nutritional psychiatry studies is to stratify the meat that was consumed into red, white and processed. These are considerations to be included in future studies that aim to explore and understand the influence of the types of meat consumed by the older adult population in Singapore on depression.

### Strengths & Limitations

The study did not categorise the different types of fish according to their taxonomic classification as it has been traditionally studied whilst collecting data. The current paper attempted to assign the taxonomic classification post hoc based on the most frequent species of the fish family found in Singapore. The different terminologies and heterogeneous multi-ethnic nature of Singapore’s languages among Singapore’s older adult population made it difficult to place the fish into their specific taxonomic classification. Cooking styles (i.e. type of oil, fried vs. steamed), method of preservation, and the different units of fish consumption were not considered as well – these are factors that have been identified as significant determinants in a recent meta-analysis [21]. The study did not take into account the type of cuisine, as wells as the type of V&F consumed by the older adults in Singapore. The study utilised the GMS-AGECAT, which does not capture information on personality characteristics, episodic depression, stress and unpleasant memories. Furthermore, the study did not explore the energy and fat intake of the proposed diet variables and problematic alcohol use. However, the study has its strengths; this includes a large sample size with a fair response rate of 65.6%, and the use of structured and validated instruments in the local languages to make a diagnosis of depression added to the rigour of the study [38].

## Conclusion

The consumption of V&F within the last 3 days, the frequent consumption of certain common types of fish, meat consumption, and PAU were associated with depression and/or subsyndromal depression. These findings support the importance for a healthy consumption of V&F, specific fish, less meat consumption, and PAU for the older adult population in Singapore. However, these are preliminary findings and require a more detailed health and nutrition survey to accurately capture local diets to help inform public dietary policies, and promote healthier dietary choices among the older adults in Singapore.

## Data Availability

Data supporting the findings is available upon request. Please contact the Principal Investigator of the study, Professor Chong Siow Ann at siow_ann_chong@imh.com.sg, for data availability.

## Abbreviations

*A. mate*: *Atule mate* / Yellow Tail
CAGE: Cut, Annoyed, Guilty, and Eye-opener Questionnaire
DHA: Docosahexaenoic Acid
*E. coioides*: *Epinephelus coioides* / Grouper
EPA: Eicosapentaenoic Acid
FFQ: Food Frequency Questionnaire
GMS-AGECAT: Geriatric Mental State-Automated Geriatric Examination for Computer Assisted Taxonomy
H. Gerrardi: Himantura Gerrardi / Stingray
*L. calcarifer*: *Lates calcarifer* / Seabass
MDD: Major Depressive Disorder
P. chinesis: Pampus chinesis / Pomfret
*P. multidens*: *Pristipomoides multidens* / Snapper
PUFA: Polyunsaturated Fatty Acids
*S. commerson*: *Scomberomorus commerson* / Mackerel
SMHS: Singapore Mental Health Study
SSD: Subsyndromal Depression
TFA: Trans-unsaturated Fats
V&F: Vegetables and Fruits
WHO: World Health Organisation
WiSE: The Well-being of the Singapore Elderly
WWF: World Wide Fund for Nature

## References

[1] World Health Organization*.* 2017*.* Depression and other common mental disorders: global health estimates. Rep. CC BY-NC-SA 3.0 IGO, World Health Org., Geneva.

[2] Valiengo Lda C, Stella F, Forlenza OV (2016). Mood disorders in the elderly: prevalence, functional impact, and management challenges. Neuropsychiatr Dis Treat.

[3] *United Nations, Department of Economic and Social Affairs, Population Division (*2017*). World Population Ageing* 2017 *- Highlights (ST/ESA/SER.A/397).*

[4] Chong SA (2012). A population-based survey of mental disorders in Singapore. Ann Acad Med Singap.

[5] Subramaniam M (2016). Prevalence of depression among older adults--results from the well-being of the Singapore elderly study. Ann Acad Med Singap.

[6] Singhal T, Kothari S (2018). The effect of docosahexaenoic acid (DHA) supplementation on experimental depression in mice. International Journal of Basic & Clinical Pharmacology.

[7] Lang UE (2015). Nutritional aspects of depression. Cell Physiol Biochem.

[8] Li N (2011). Risk factors for depression in older adults in Beijing. Can J Psychiatr.

[9] Jacka FN (2014). Dietary patterns and depressive symptoms over time: examining the relationships with socioeconomic position, health behaviours and cardiovascular risk. PLoS One.

[10] Jacka FN (2017). Nutritional psychiatry: where to next?. EBioMedicine.

[11] Zhang Y (2017). Is meat consumption associated with depression? A meta-analysis of observational studies. BMC Psychiatry.

[12] Sánchez-Villegas A (2009). Association of the mediterranean dietary pattern with the incidence of depression: the seguimiento universidad de Navarra/university of Navarra follow-up (sun) cohort. Arch Gen Psychiatry.

[13] Sanchez-Villegas A (2006). Mediterranean diet and depression. Public Health Nutr.

[14] Nanri A (2010). Dietary patterns and depressive symptoms among Japanese men and women. Eur J Clin Nutr.

[15] Quirk SE (2013). The association between diet quality, dietary patterns and depression in adults: a systematic review. BMC Psychiatry.

[16] Awaworyi Churchill S, Farrell L (2017). Alcohol and depression: evidence from the 2014 health survey for England. Drug Alcohol Depend.

[17] Boden JM, Fergusson DM (2011). Alcohol and depression. Addiction.

[18] Caputo F (2012). Alcohol use disorders in the elderly: a brief overview from epidemiology to treatment options. Exp Gerontol.

[19] Ong CW (2016). Screening for drinking problems in the elderly in Singapore using the CAGE questionnaire. Ann Acad Med Singap.

[20] Matsuoka YJ (2017). Dietary fish, n-3 polyunsaturated fatty acid consumption, and depression risk in Japan: a population-based prospective cohort study. Transl Psychiatry.

[21] Li F, Liu X, Zhang D (2016). Fish consumption and risk of depression: a meta-analysis. J Epidemiol Community Health.

[22] Wolniczak I (2017). Fruits and vegetables consumption and depressive symptoms: a population-based study in Peru. PLoS One.

[23] Bishwajit G (2017). Association between depression and fruit and vegetable consumption among adults in South Asia. BMC Psychiatry.

[24] Hibbeln JR (1998). Fish consumption and major depression. Lancet.

[25] Lai JS (2014). A systematic review and meta-analysis of dietary patterns and depression in community-dwelling adults. Am J Clin Nutr.

[26] Dey S, Misra S, Homechoudhuri S (2017). Reviewing nutritional quality of small freshwater fish species. American Journal of Food and Nutrition.

[27] Mohanty BP (2016). DHA and EPA content and fatty acid profile of 39 food fishes from India. Biomed Res Int.

[28] Özogul Y, Özogul F (2007). Fatty acid profiles of commercially important fish species from the Mediterranean, Aegean and black seas. Food Chem.

[29] Özogul Y, Özogul F, Alagoz S (2007). Fatty acid profiles and fat contents of commercially important seawater and freshwater fish species of Turkey: a comparative study. Food Chem.

[30] Muhamad NA, Mohamad J (2012). Fatty acids composition of selected Malaysian fishes. Sains Malaysiana.

[31] Osman F (2007). Fatty acid profiles of fin fish in Langkawi Island, Malaysia. Journal of Oleo Science.

[32] Tsai AC, Chang TL, Chi SH (2012). Frequent consumption of vegetables predicts lower risk of depression in older Taiwanese - results of a prospective population-based study. Public Health Nutr.

[33] Reynolds E (2006). Vitamin B12, folic acid, and the nervous system. The Lancet Neurology.

[34] Payne ME (2012). Fruit, vegetable, and antioxidant intakes are lower in older adults with depression. J Acad Nutr Diet.

[35] *Agri-Food & Veterinary Authority of Singapore (AVA).* 2018*.* Annual Report 2017/18. Retrieved from https://www.sfa.gov.sg/docs/default-source/publication/annual-report/ava-ar-2017-18.pdf*.* Accessed 24 May 2019*.*

[36] Kwek, A.*, Seafood Report 2017. In: Singapore: Seafood Report. 2017.* https://www.fas.usda.gov/data/singapore-seafood-report-2017*.* Accessed 23 May 2019*.*

[37] *World Health Organization.* 2014*. Singapore Alcohol Consumption: Levels and Patterns. Retrieved from* https://www.who.int/substance_abuse/publications/global_alcohol_report/profiles/sgp.pdf*.* Accessed 24 May 2019*.*

[38] Subramaniam M (2015). Prevalence of dementia in people aged 60 years and above: results from the WiSE study. J Alzheimers Dis.

[39] Fauziana R (2016). Body mass index, waist-hip ratio and risk of chronic medical condition in the elderly population: results from the well-being of the Singapore elderly (WiSE) study. BMC Geriatr.

[40] Abdin E (2018). A non-parametric item response theory evaluation of the CAGE instrument among older adults. Subst Use Misuse.

[41] Kua EH (1992). A community study of mental disorders in elderly Singaporean Chinese using the GMS-AGECAT package. Aust N Z J Psychiatry.

[42] Lytle JS, Lytle TF (1994). Fatty acid and cholesterol content of sharks and rays. J Food Compos Anal.

[43] Burgess KB (2018). Novel signature fatty acid profile of the giant manta ray suggests reliance on an uncharacterised mesopelagic food source low in polyunsaturated fatty acids. PLoS One.

[44] Kris-Etherton PM (2000). Polyunsaturated fatty acids in the food chain in the United States. Am J Clin Nutr.

[45] Neupane SP (2014). High frequency and intensity of drinking may attenuate increased inflammatory cytokine levels of major depression in alcohol-use disorders. CNS Neurosci Ther.

[46] Szabo G, Mandrekar P (2009). A recent perspective on alcohol, immunity, and host defense. Alcohol Clin Exp Res.

[47] Clegg A (2013). Frailty in elderly people. Lancet.

[48] Kiecolt-Glaser JK, Glaser R (2002). Depression and immune function. J Psychosom Res.

[49] Sánchez-Villegas A (2013). Mediterranean dietary pattern and depression: the PREDIMED randomized trial. BMC Med.

[50] Sánchez-Villegas A (2011). Dietary fat intake and the risk of depression: the SUN project. PLoS One.

[51] Popa T, Ladea M (2012). Nutrition and depression at the forefront of progress. Journal of medicine and life.

